# Long‐Term Outcomes of Triple Cannulated Compression Screws Combined With Bone Graft Sleeve Parallel Implantation of DBM Crunch Internal Fixation for the Treatment of Femoral Neck Fractures in Middle‐Aged and Young Adults

**DOI:** 10.1111/os.70169

**Published:** 2025-10-09

**Authors:** Peiyuan Wang, Zhiang Zhang, Zihang Zhao, Ziping Li, Lin Liu, Kuo Zhao, Lin Jin, Wei Chen, Shiqiang Zhang, Zhiyong Hou

**Affiliations:** ^1^ Orthopaedic Research Institute of Hebei Province Shijiazhuang Hebei People's Republic of China; ^2^ Department of Orthopaedic Surgery The Third Hospital of Hebei Medical University Shijiazhuang Hebei People's Republic of China; ^3^ NHC Key Laboratory of Intelligent Orthopaedic Equipment (The Third Hospital of Hebei Medical University) Shijiazhuang People's Republic of China

**Keywords:** bone graft sleeve, cannulated compression screws, demineralized bone matrix crunch, femoral neck fractures, young and middle‐aged

## Abstract

**Objective:**

If the appropriate internal fixation surgical method is not adopted for femoral neck fractures in young people, it may lead to serious consequences such as poor fracture healing and femoral head necrosis, affecting the quality of life and working ability of young people. Therefore, it is crucial to conduct in‐depth research on the internal fixation surgical methods. This study compared the therapeutic effects of triple cannulated screws combined with a bone graft sleeve for parallel implantation of DBM Crunch internal fixation (CCSBGS) and cannulated compression screws (CCS).

**Methods:**

Medical records on the young and middle‐aged patients with femoral neck fracture treated with two different internal fixation methods from January 2020 to June 2023 were collected and retrospectively analyzed in the Trauma Emergency Center of the Third Hospital of Hebei Medical University. Two internal fixation groups are: CCSBGS group with 50 patients, 35 males and 15 females, aged (42.44 ± 14.07) years; CCS group with 80 males and 39 females, aged (41.5 ± 13.48) years. This study compared the outcome measures of two groups of patients, including Garden alignment index, Operation duration time, Intraoperative blood loss, Length of hospital stay, Postoperative complications, Femoral neck shortening, Postoperative ambulation time, Walking with sticks, Barthel score, and Harris score.

**Results:**

There was a statistically significant difference in blood loss between the CCS group and the CCSBGS group; at the same time, the amount of bleeding in the CCS group was lower than that in the CCSBGS group (*p* < 0.01). During the follow‐up period, there was a statistically significant difference in the incidence of osteonecrosis of the femoral head among the two groups (*p* < 0.05), 20 patients in the CCS group and 2 patients in the CCSBGS group developed osteonecrosis of the femoral head. At the last follow‐up, the average degree of femoral neck shortening in the CCSBGS group [(0.49 ± 0.28) cm] was significantly lower than that in the CCS group [(0.87 ± 0.35) cm] (*p* < 0.05). Meanwhile, the postoperative ambulation time of the CCSBGS group is earlier than that of the CCS group (*p* < 0.05). In addition, the CCSBGS group had the highest Barthel scores [(95.50 ± 2.90)] (*p* < 0.05). The average Harris score in the CCSBGS group [(92.52 ± 2.41)] was higher than that in the CCS group [(90.47 ± 2.88)] (*p* < 0.05).

**Conclusions:**

Compared with CCSBGS and CCS, CCSBGS shows better efficacy in terms of quicker return to weight‐bearing activities, preservation of femoral neck length, reduction of the rate of osteonecrosis of the femoral head, and overall enhancement of hip function.

## Graphical abstract

This study compared the therapeutic effects of two internal fixation methods, including triple cannulated screws combined with a bone graft sleeve for parallel implantation of DBM Crunch internal fixation (CCSBGS), and cannulated compression screws (CCS). Fracture reduction and CCS fixation were the same as those in the CCS group. In addition, a guide pin was placed in parallel at the center of the 3 CCS, and it was confirmed that the guide pin passed through the fracture end. A suitable bone tunnel was established by expanding the central guide pin 0.5 cm to the subchondral bone. A 5 mL volume of Demineralized Bone Matrix Crunch (DBM Crunch) was verified to pass through the fracture line using fluoroscopy, guided by the BGS.

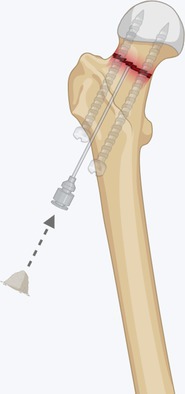

**Abbreviations**
BMI: body mass index
CCS: cannulated compression screws
CCSBGS: cannulated screws combined with a bone graft sleeve
DBM: malleable demineralized bone matrix crunch
GAI: garden alignment index
SD: mean ± standard deviation

## 1 Introduction

Femoral neck fractures are often diagnosed in the field of orthopedics and are frequently associated with a notably elevated risk of both disability and mortality [1, 2]. In the context of younger patients possessing enhanced mobility, internal fixation presents several advantages, including diminished trauma, preservation of the integrity of the femoral head, and enhanced postoperative hip joint functionality. Additionally, patients may require one or more revision surgeries for artificial hip joints owing to infection, aseptic loosening, or other factors. Nevertheless, the selection of internal fixation continues to pose a notably complex decision‐making challenge in the field of traumatic orthopedics [3, 4, 5]. The existing internal fixation methods include the use of cannulated compression screws (CCS). Triple cannulated screw fixation stands as the traditional surgical approach. The triangular distribution constructed in this technique can create a three‐dimensional structure with skeleton and bone tissue, reducing stress on the rotation of the femoral head. This method amplifies the compressive stress applied between the fractured ends both during and following the surgical procedure. This, in turn, fosters a tight and intimate connection between the fractured ends, thereby expediting the process of fracture healing. However, there is no correlation among the three cannulated screws, and the screw position can be easily influenced by subjective and objective factors associated with the surgeon. As a result, its ability to resist vertical shear and torsion is poor, potentially leading to the loosening and displacement of the fracture end, femoral head necrosis and nonunion, and femoral neck shortening [6]. In recent years, as a result of advancements in the social economy and increasing patient expectations for surgical outcomes, the significance of Malleable Demineralized Bone Matrix Crunch (DBM Crunch) in the domain of traumatic orthopedics has been progressively acknowledged and appreciated. Therefore, improvements were made on the described basis, leading to the adoption of a treatment approach involving the use of triple cannulated screws combined with a bone graft sleeve (CCSBGS) for parallel implantation of DBM Crunch internal fixation. This approach was employed in the management of femoral neck fractures in middle‐aged and young adults, yielding satisfactory results.

Thus, the aim of the present study mainly consists of three parts: (i) This retrospective study compares CCSBGS with conventional CCS for femoral neck fractures in young adults, evaluating Garden reduction, operative metrics, complications, functional scores, and daily living to confirm CCSBGS's superior healing, lower morbidity, and better hip function. (ii) Operative complexity, trauma, functional recovery, cost, and learning curve are analyzed to assess the feasibility, safety, and scalability of CCSBGS, setting the stage for larger multicenter randomized controlled trials. (iii) Focusing on the DBM Crunch component, the work elucidates how osteoinductive allograft promotes bone integration, modulates femoral head stress, and reduces osteonecrosis risk, providing a mechanistic basis for next‐generation bone‐graft techniques.

## 2 Materials and Methods

### 2.1 Patients

In this retrospective study, data were collected from 169 patients with femoral neck fractures who underwent treatment at our hospital between January 2020 and June 2023. The patients were divided into two groups in total. Among them, there were 119 patients in the CCS group and 50 patients in the CCSBGS group. The patients were followed up for at least 15 months. The study protocol was performed in compliance with the Helsinki Declaration. All patients participated in the study voluntarily and signed the informed consent to participate in the study. This study was approved by the Medical Ethics Committee of the Third Hospital of Hebei Medical University (G2020‐002‐1).

### 2.2 Inclusion Criteria and Exclusion Criteria

The inclusion criteria were as follows: (1) individuals aged 18 to 65 years with recent femoral neck fractures, (2) patients who exhibited independent ambulation before sustaining the injury, (3) patients who underwent treatment with either CCS or CCSBGS, and (4) individuals who underwent a follow‐up period of at least 15 months. The exclusion criteria were as follows: (1) individuals with concurrent fractures or congenital hip dysplasia, (2) cases involving pathological fractures, (3) instances of open fractures, and (4) patients with cognitive impairments.

### 2.3 The Description of BGS

**FIGURE 1:**
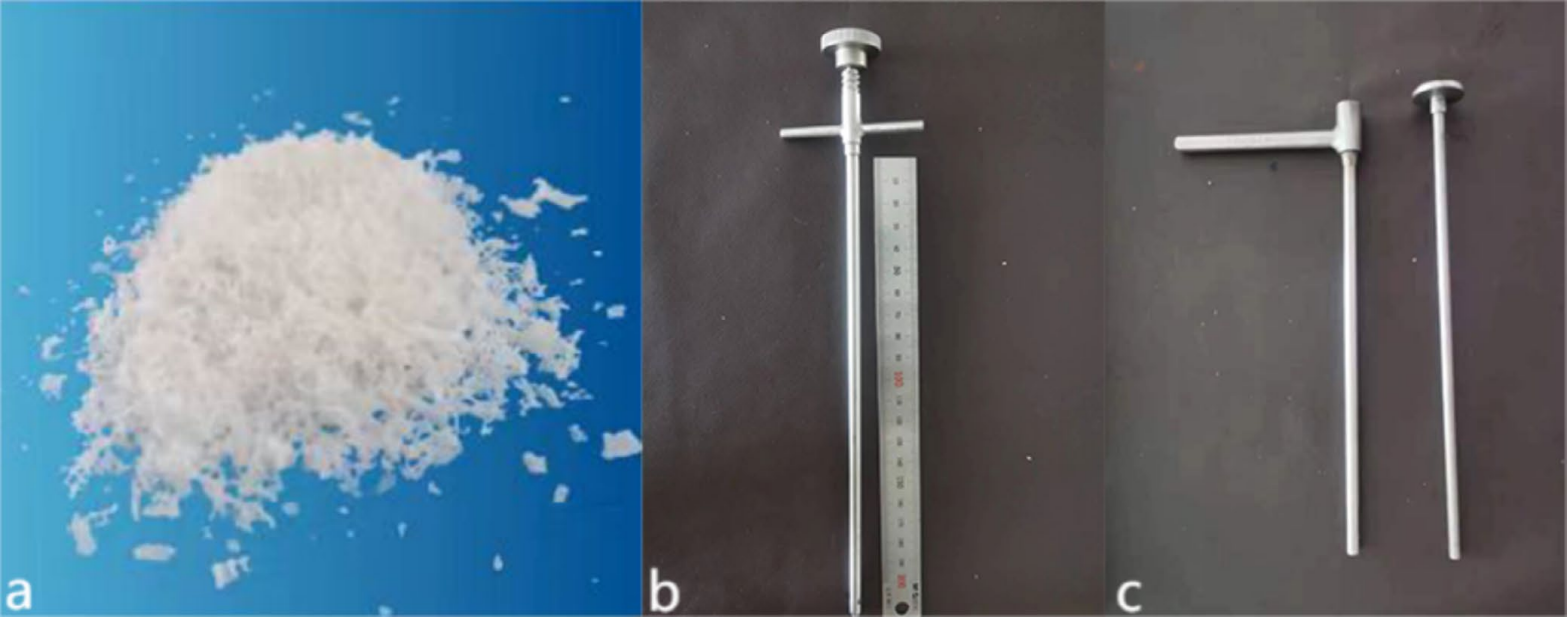
(a) Malleable demineralized bone matrix crunch. (b, c) Bone graft sleeve.

**FIGURE 2:**
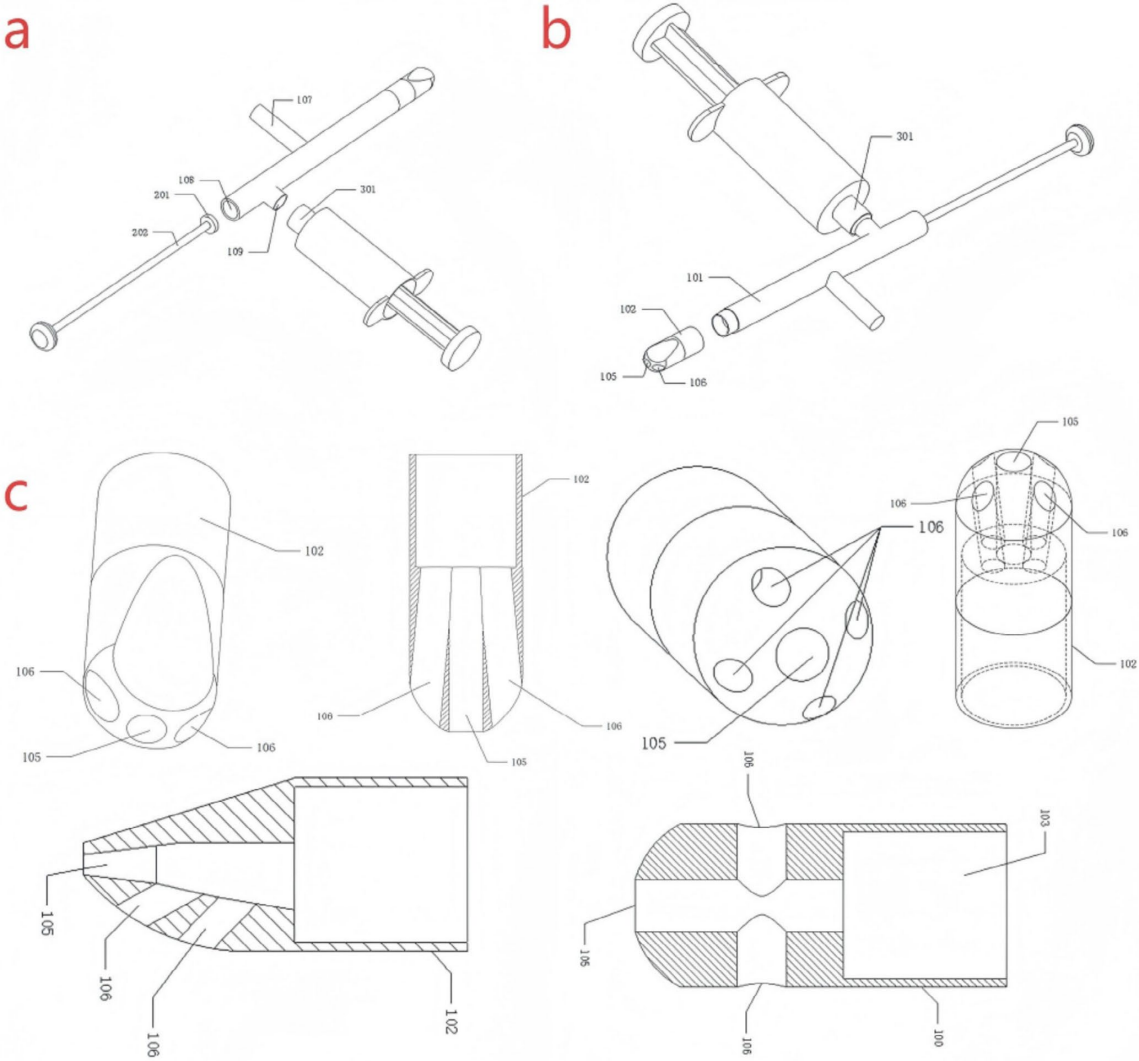
The blueprint of BGS (a, b) A general view of BGS. (c) Close‐up of the BGS head. *(100) Filling a pipe. (101) First pipe section. (102) Second pipe section. (103) Main body channel. (104) First branch channel. (105) First filling hole. (106) Second filling hole. (107) Grip. (108) Working hole. (109) Injection hole. (201) Piston. (202) Push rod. (301) Syringe nipple.

The device primarily comprises the following components: a filling pipe, which features a working hole at one end and multiple filling holes in various directions at the other end. Within the hollow channel of the filling pipe, there exists a primary channel running parallel to the axis of the filling pipe. One end of this primary channel is linked to the working hole, while the other end connects to numerous branch channels. Each branch channel is associated with a filling hole. Among these multiple filling holes, the first filling hole stands out, and the branch channel connecting the first filling hole to the primary channel is referred to as the first branch channel. The working hole, the main channel, the first branch channel, and the first filling hole are arranged coaxially. The working piston enters the main channel through the working hole and can move along the main channel to squeeze the bone powder in the main channel. This action results in the bone powder being expelled from the filling tube through each filling hole via the branch channel. The device can improve the accuracy of the filling position and the filling efficiency of the bone powder (Figures 1 and 2).

DBM Crunch is derived from donor cortical bone that has been screened for infection, decalcified in 0.6 N HCl for 5 to 7 days, de‐fatted, de‐antigen‐treated, freeze‐dried, γ‐sterilized, and milled to 50 to 400 μm white granules with < 1% residual calcium and preserved BMPs. It is commercially supplied by Shanghai Yapeng, and is stored at 2°C to 8°C for up to 24 months.

### 2.4 The Operation Process

Patients received intravenous antibiotics 30 min before surgery. Following successful anesthesia induction, the patient was placed in the supine position, and the surgical field was disinfected with a 2.5% tincture of iodine and 75% alcohol. Sterile towels were then applied. For the surgical procedure, the patient was placed on an orthopedic traction bed, and the lower limbs were abducted and internally rotated. Fluoroscopy was employed to assist in the reduction of fractures, and all surgical procedures were conducted collaboratively by skilled and experienced medical practitioners.

#### 2.4.1 CCS Group

Traction reduction of femoral neck fracture under fluoroscopy of C‐arm x‐ray machine. After satisfactory resetting according to the surgeon's experience, three CCS guide pins were inserted in parallel under fluoroscopic guidance in an inverted “triangle” shape. After measuring the length, three CCSs were screwed in successively to complete the compression fixation of the fracture end. The tip of each CCS was 0.5 cm from the subchondral bone. Following fluoroscopy, the incision site was thoroughly cleansed and sutured.

#### 2.4.2 CCSBGS Group

**FIGURE 3:**
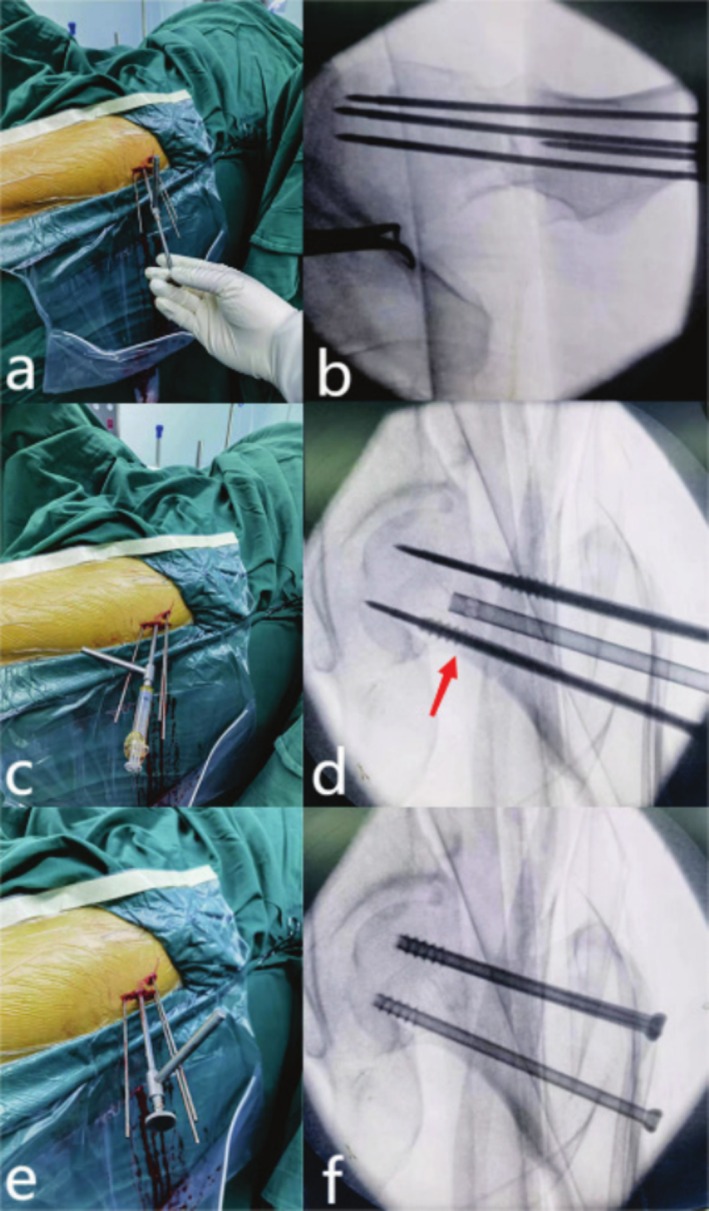
The operation process of CCSBGS (a, b) Insert the BGS along the central guide pin. (c, d) Push the DBM into the channel. (e, f) Screw three cannulated compression screws.

Fracture reduction and CCS fixation were the same as those in the CCS group. In addition, a guide pin was placed in parallel at the center of the 3 CCS, and it was confirmed that the guide pin passed through the fracture end. A suitable bone tunnel was established by expanding the central guide pin 0.5 cm to the subchondral bone. A 5 mL volume of Demineralized Bone Matrix Crunch (DBM Crunch) was verified to pass through the fracture line using fluoroscopy, guided by the BGS. Subsequently, the depth was measured, and three screws were inserted with consistent pressure. The remaining steps of the procedure were identical to those performed in the CCS group (Figure 3).

### 2.5 Perioperative Management

Patients were prophylactically administered first‐generation cephalosporins 0.5 h before and after surgery. The decision to use low‐molecular‐weight heparin sodium to prevent lower extremity venous thrombosis was made based on the VTE score before and immediately after surgery. Following surgery, the affected hip joint was placed in an abduction‐neutral position, and local cold therapy was administered to reduce lower limb edema. On the first day post‐surgery, a rehabilitation therapist provided guidance to patients to initiate movements of the toe and ankle joints. They also instructed patients to engage in ankle pump training, execute isometric contractions of the quadriceps and gluteus maximus muscles, and provided assistance for hip and knee flexion exercises. These interventions aimed to prevent pulmonary infections. Patients with osteoporosis were treated with calcium, vitamin D, and calcitonin. After discharge, the patients were routinely administered oral anticoagulants and topical analgesic plasters to alleviate pain. Partial weight‐bearing training was performed as the affected limb recovered, with weight‐bearing walking allowed for 3 to 6 months after bone healing. Radiographs were reviewed within 3 days post‐surgery, and follow‐up radiographs were conducted every month for the first 6 months until healing. After the first year, radiographic assessments were conducted at 3 and 6‐month intervals.

### 2.6 Clinical Outcome Indicators

Patients' medical records were collected from the hospital's electronic medical record database and imaging system. General information included gender, age, smoke history, alcohol history, BMI, mechanism of injury, Garden type, Pauwels type, comorbidities (Hypertension, Coronary heart disease, Diabetes), and time from injury to operation. Hospitalization and operation indexes included Garden alignment index (GAI), operation duration time, intraoperative blood loss, and length of hospital stay. Following the surgical procedure, various parameters were meticulously documented, including complications (osteonecrosis of the femoral head and screw failure). Additionally, data on femoral neck shortening, postoperative ambulation time, the need for walking aids (e.g., sticks), as well as Barthel and Harris scores were collected. Evaluation of femoral head necrosis was based on the criteria outlined by Slobogean et al. which involved observing the segmental collapse of the femoral head or translucent subchondral areas on radiographs [7]. Femoral neck shortening was measured using a previously described method, with standard pelvic anteroposterior radiographs and a known screw diameter correction magnification [8]. Measurements were conducted three times by the same evaluator, and the average value was calculated for each parameter. Assessment of hip function and the ability to carry out daily activities were performed no sooner than 6 months post‐surgery, utilizing the Harris score and Barthel score, respectively [9, 10].

### 2.7 Statistical Analysis

The Shapiro–Wilk test was used to conduct the normality test on the continuous variables. The measurement data conforming to the normal distribution were expressed as mean ± standard deviation (SD), and the two‐independent sample *t*‐test was used for comparison between groups. The measurement data that do not conform to the normal distribution are expressed as M (Q_1_, Q_3_), and the non‐parametric Mann–Whitney U test is used. It is worth noting that all the data in this study conform to the normal distribution, so they are all represented by mean ± standard deviation (SD). Categorical variables were analyzed using the chi‐squared test or Fisher's exact probability method. All statistical analyses were performed using SPSS (version 26.0; IBM, Armonk, NY, USA). Statistical significance was set at *p* < 0.05.

## 3 Results

### 3.1 Comparison of the General Information Between the Two Groups

Among all 586 middle‐aged and young patients with femoral neck fracture admitted to our hospital between January 2020 and June 2023, only 169 patients were included in this study, and they were assigned to two different groups: the CCS group (*n* = 119) and CCSBGS group (*n* = 50). There were no significant differences in gender, age, smoking history, alcohol history, BMI, mechanism of injury, Garden type, Pauwels type, comorbidities, or time from injury to operation between the groups (Table 1).

**TABLE 1:** Comparison of the general information between the two groups.

	CCS	CCSBGS	*p*
Cases	119	50	—
Gender (male/female)	80/39	35/15	0.72
Age (years)	41.5 ± 13.48	42.44 ± 14.07	0.68
Smoke history			0.72
Yes	80	35	
No	39	15	
Alcohol history			0.85
Yes	60	24	
No	59	26	
BMI (kg/m^2^)	23.69 ± 3.14	23.38 ± 3.84	0.58
Mechanism of injury			0.90
Fall down	39	17	
High falling	40	18	
Traffic injury	40	15	
Garden type			0.87
II	45	21	
III	24	9	
IV	50	20	
Pawels type			0.71
I	32	11	
II	20	12	
III	28	12	
IV	39	15	
Comorbidities			
Hypertension			0.86
Yes	13	5	
No	106	45	
Coronary heart disease			0.71
Yes	9	3	
No	110	47	
Diabetes			0.62
Yes	7	2	
No	112	48	
Time from injury to operation (days)	8.49 ± 3.52	9.54 ± 3.7	0.08

### 3.2 Comparison of Hospitalization and Operation Between the Two Groups

The operation time of patients treated with CCS was longer than that of those treated with CCSBGS (90.5 ± 19.63 min, and 83.79 ± 21.87 min, *p* < 0.05). However, no significant differences were observed in GAI among the CCS and CCSBGS groups. The intraoperative blood loss was 84.75 ± 32.59 mL in the CCS group and 123.1 ± 46.98 mL in the CCSBGS group. A statistically significant difference in blood loss was observed among the two groups, with the CCSBGS group displaying a higher amount of bleeding (*p* < 0.01). However, there was no significant difference in the length of hospital stay among the CCS group and the CCSBGS group (Table 2).

**TABLE 2:** Comparison of hospitalization and operation between the two groups.

	CCS	CCSBGS	*p*
Garden alignment index (GAI)			0.60
I	110	45	
II	9	5	
Operation duration time (min)	83.79 ± 21.87	90.5 ± 19.63	0.06
Intraoperative blood loss (mL)	84.75 ± 32.59	123.1 ± 46.98	< 0.01
Length of hospital stay (days)	10.6 ± 1.69	10.4 ± 1.6	0.47

### 3.3 Comparison of Postoperative Complications and Function Recovery Between the Two Groups

**FIGURE 4:**
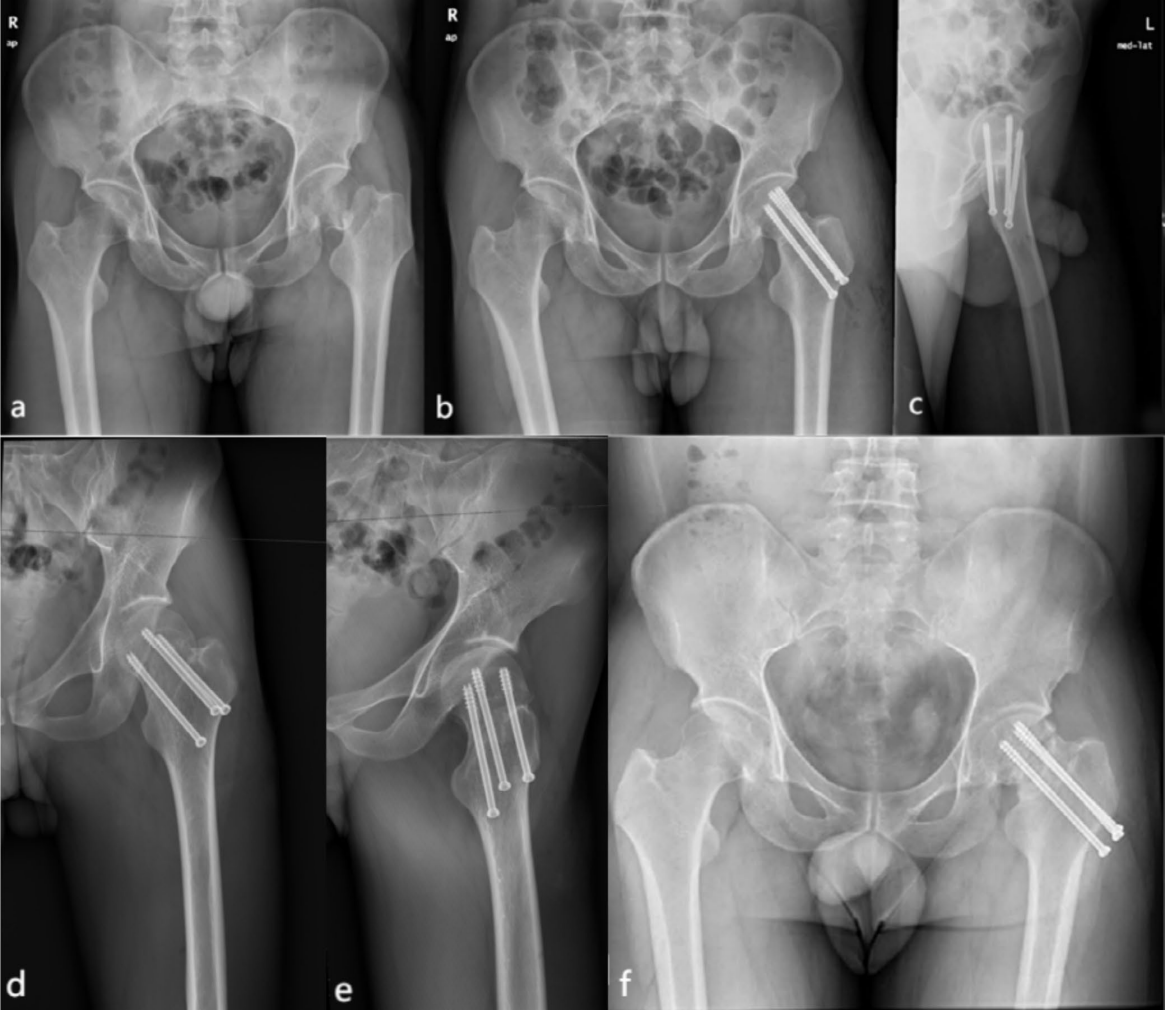
A 47‐year‐old male patient with a left femoral neck fracture was treated with CCS. (a) Preoperative X‐ray image. (b, c) Postoperative x‐ray images. (d, e) Postoperative 6‐month follow‐up radiographs. (f) Postoperative 16‐month follow‐up radiographs showing femoral head necrosis.

**FIGURE 5:**
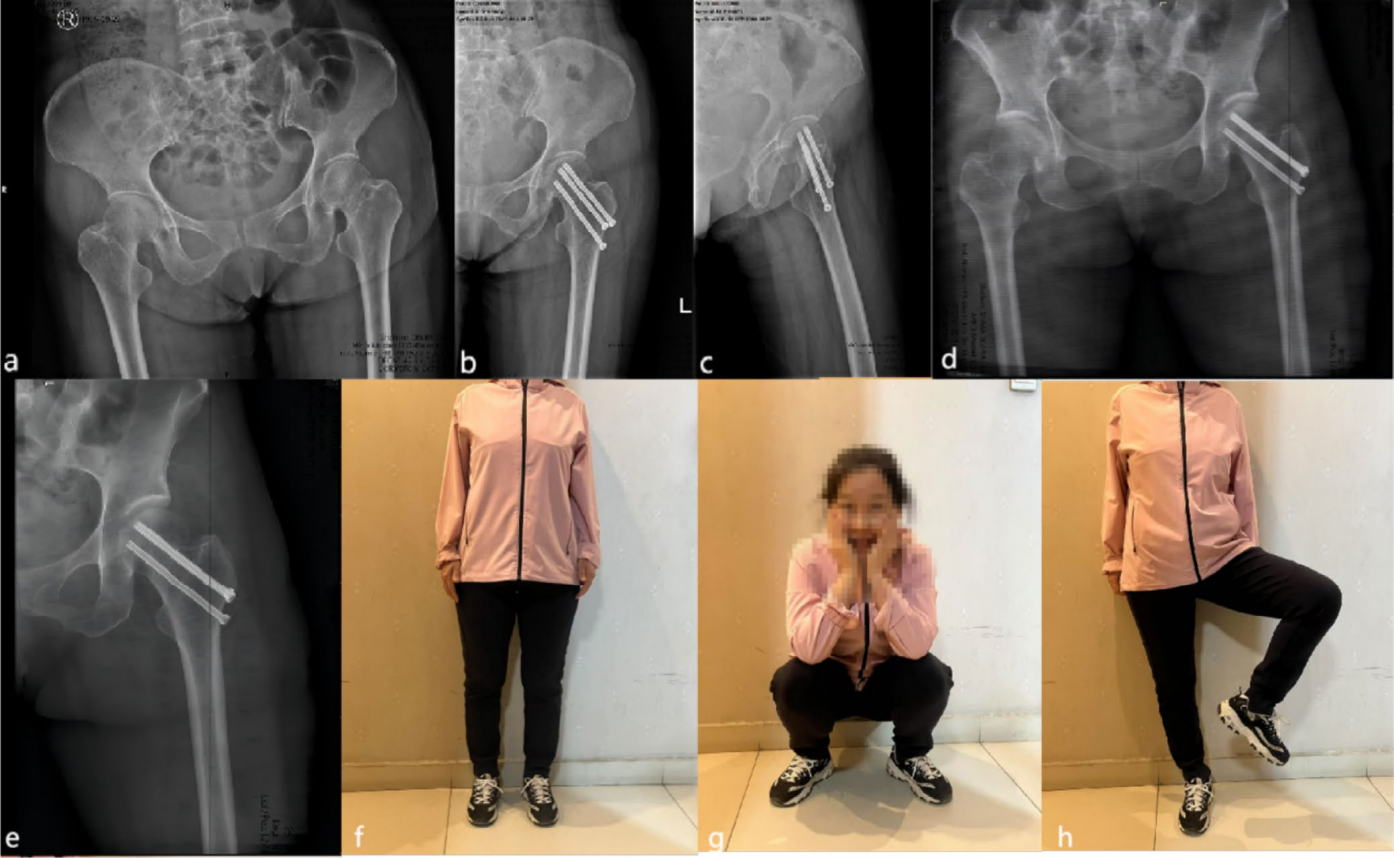
A 58‐year‐old female patient with a left femoral neck fracture was treated with CCSBGS. (a) Preoperative x‐ray image. (b, c) Postoperative x‐ray images. (d) Postoperative 7‐month x‐ray image. (e) Postoperative 15‐month x‐ray image showing that the femoral neck fracture recovers well. (f, g, h) The patient recovered with a normal gait, good function, and no discomfort such as pain. The Harris score was 94.

At the last follow‐up after operation, in the CCS group, osteonecrosis of the femoral head occurred in 20 cases (16.8%), and in the CCSBGS group, 2 patients (4.0%) experienced osteonecrosis of the femoral head. There was a significant difference in the complications among the CCS group and the CCSBGS group (*p* < 0.05) (Table 3). However, the incidence of internal fixation failure was not statistically significant between the two groups. Among the 119 patients in the CCS group, there was an average femoral neck shortening of 0.867 ± 0.348 cm. Meanwhile, in the CCSBGS group, the average femoral neck shortening measured 0.489 ± 0.277 cm. There was a significant difference in the femoral neck shortening among the two groups (*p* < 0.01) (Table 3). At the same time, the postoperative ambulation time of the CCSBGS group is earlier than that of the CCS group (*p* < 0.05). The postoperative Barthel scores were 88.40 ± 6.89 and 95.50 ± 2.90 in the CCS and CCSBGS groups. The CCSBGS group showed the highest Barthel score (*p* < 0.01) (Table 3). In addition, the postoperative Harris scores were 90.47 ± 2.88 and 92.52 ± 2.41 in the CCS and CCSBGS groups, respectively. Notably, the CCSBGS group demonstrated the highest Harris score (*p* < 0.01), as indicated in Table 3. However, there was no statistically significant difference in the need for walking aids among the two groups (Figures 4 and 5).

**TABLE 3:** Comparison of postoperative complications and functional recovery between the two groups.

	CCS	CCSBGS	*p*
Complication			
Osteonecrosis of the femoral head			0.02
Yes	20	2	
No	99	48	
Internal fixation failure			0.49
Yes	11	3	
No	108	47	
Femoral neck shortening (cm)	0.87 ± 0.35	0.49 ± 0.28	< 0.01
Postoperative ambulation time (weeks)	17.31 ± 3.97	11.38 ± 3.02	< 0.01
Walking with sticks			0.48
Yes	40	14	
No	79	36	
Barthel score	88.40 ± 6.89	95.50 ± 2.90	< 0.01
Harris score	90.47 ± 2.88	92.52 ± 2.41	< 0.01

## 4 Discussion

At baseline, there were no significant differences in patient characteristics between the two groups (*p* > 0.05). However, a statistically significant difference was observed in blood loss between the CCS group and the CCSBGS group (*p* < 0.01), with the CCS group experiencing less bleeding compared to the CCSBGS group. During the follow‐up period, the incidence of osteonecrosis of the femoral head differed significantly between the two groups (*p* < 0.05), with 20 cases in the CCS group and only 2 cases in the CCSBGS group. At the final follow‐up, the average degree of femoral neck shortening was significantly lower in the CCSBGS group than in the CCS group (*p* < 0.05). Additionally, the CCSBGS group had an earlier postoperative ambulation time of (11.38 ± 3.02) weeks compared to the CCS group (*p* < 0.05). The CCSBGS group also achieved the highest Barthel scores (*p* < 0.05) and had a significantly higher average Harris score than the CCS group (*p* < 0.05).

### 4.1 The Mechanism of Action and Potential Advantages of CCSBGS

DBM, produced by acid extraction of pulverized allograft [11, 12, 13, 14], is the only clinically available osteoinductive material, usually formulated as a powder or carrier‐added putty [15, 16]. Mixed with autograft or marrow, it expands graft volume and equals iliac‐crest fusion rates using only one‐third autograft [17, 18]. DBM Crunch efficacy is proven in spine and long‐bone fusion [17, 19, 20, 21], yet data in young to middle‐aged femoral neck fractures remain scarce.

### 4.2 The Clinical Application Value and Feasibility of CCSBGS

It has been documented that the prevalence of postoperative complications, specifically avascular necrosis of the femoral head and bone nonunion, among young adults afflicted with femoral neck fractures, can be as elevated as 86% [22]. Even early surgery (< 12 h) does not reduce the risk of nonunion or avascular necrosis of the femoral head [23]. Further, G.P. Lobogean conducted a comprehensive analysis of significant complications following triple CCS internal fixation in patients below the age of 60. The study revealed that the incidence of reoperation after CCS internal fixation for femoral neck fractures was approximately 20%. Among these cases, femoral head necrosis and bone nonunion emerged as the most prevalent complications necessitating reoperation [4]. However, in the present study, findings were made that the incidence of osteonecrosis of the femoral head in the CCSBGS group was lower compared to the CCS group. Li et al. observed that decalcified bone matrix exhibited favorable chondrogenic activity. In their study, there was an increased expression of type II collagen within the matrix following co‐culture with chondrocytes [24]. Moreover, in the field of bone tissue engineering, Mauney discovered that three‐dimensional partial decalcified bone exhibited stronger bone‐inductive properties compared to other synthetic materials [25]. It also enhanced the differentiation ability of human bone mesenchymal stem cells, thereby stimulating osteogenesis and facilitating the process of fracture healing. Finally, after a period of biological healing, the DBM Crunch developed a supportive effect characterized by a substantial contact area beneath the cartilage. Simultaneously, the elastic modulus of DBM Crunch closely matches that of the femoral head tissue. This alignment helps prevent stress concentration and the formation of necrotic fracture zones, ultimately contributing to the repair of necrotic regions within the femoral head and preventing femoral head collapse subsequent to necrosis.

### 4.3 The Clinical Efficacy of Different Internal Fixation Methods

Parallel screws as well as dynamic or sliding hip screws allow fracture fragments to slide against each other along the implant and therefore allow compression when subjected to axial loading forces during weight‐bearing. The biological rationale behind this sliding concept involves the promotion of fracture healing by compressing fracture fragments. However, this fixation principle may lead to femoral neck shortening and alter the moment arm of the hip abductor muscles, potentially negatively impacting the patient's physical function [26]. In our study, we observed that the extent of postoperative femoral neck shortening in the CCSBGS group was less pronounced compared to the CCS group. These findings highlight that the use of allograft bone can mitigate femoral neck shortening without compromising the sliding pressure, ultimately benefiting early ambulation and the overall functional recovery of patients. Further, in the evaluation of postoperative Harris scores and Barthel scores, the CCSBGS group exhibited a significant advantage over the CCS group, providing additional support for the aforementioned conclusion.

Inevitably, the incorporation of bone grafting procedures will extend both the duration of the surgical operation and the amount of blood lost during the procedure. Consequently, it was observed that the surgical duration and intraoperative blood loss in the CCSBGS group exceeded those in the CCS group. Nonetheless, the extended surgical duration associated with the inclusion of bone grafting procedures is not significantly pronounced, and the postoperative advantages outweigh the minor increase in operation time, rendering it still within acceptable limits. Moreover, for young and middle‐aged patients, the level of blood loss resulting from bone grafting remains within acceptable boundaries [27, 28]. Meanwhile, when compared to the traditional surgical approach, CCSBGS did not introduce additional complexity, and the learning curve associated with it was found to be relatively short. At the last follow‐up, 33% and 28% of the patients in the CCS group and CCSBGS group still needed crutches to walk, respectively, but there was no significant difference between the two groups (*p* > 0.05). This could be related to the short follow‐up time of the present study or the psychological factors of patients.

### 4.4 Surgical Tips for CCSBGS

(i) After standard CCS fixation, insert a central guide pin parallel to the three screws, ensuring it crosses the fracture. (ii) Enlarge this track 0.5 cm to subchondral bone, then under fluoroscopy deliver 5 mL DBM Crunch through the fracture line via the BGS sleeve. (iii) Measure depth, insert the three screws with uniform compression, and complete the surgery as same as CCS.

### 4.5 Limitations and Strengths

This study, through a large sample size of young patients with femoral neck fractures, multi‐dimensionally compared the clinical efficacy of two internal fixation methods, CCSBGS and CCS, and obtained comprehensive and in‐depth results in terms of surgery‐related indicators, postoperative complications, and functional recovery. Focusing on the youth group, it provides clinicians with more effective treatment options and optimizes the treatment plan for femoral neck fractures in young people. Meanwhile, the application effects of the new internal fixation techniques CCSBGS and DBM Crunch bone graft materials were evaluated, and their mechanisms of action in femoral neck fractures in young people were explored, providing a reference for the future development of new bone graft materials. The data collection is detailed and comprehensive, and the analysis is scientific and reliable, ensuring the scientific and reliable nature of the research results, which can provide strong evidence for clinical practice.

The present study has several limitations. First, the retrospective design may have introduced a selection bias, as the method of internal fixation was selected based on clinical experience. A randomized, multicenter prospective study should be conducted to improve the reliability of the present findings. Second, the CCSBGS group had a smaller sample size, and the short clinical application time of the CCSBGS meant that the average follow‐up time for both groups was limited. Third, at present, there is currently no standardized study addressing bone mass, depth, and bone tunnel diameter in bone graft procedures. Further research is warranted to establish appropriate guidelines and parameters in this regard. Fourth, during the measurement of the femoral neck shortening, non‐standard patient positioning during radiography may have affected the measured values.

## 5 Conclusion

In summary, the CCSBGS approach demonstrated superior outcomes in the management of femoral neck fractures when compared to the CCS. These results encompass a quicker return to weight‐bearing activities, preservation of femoral neck length, reduction of the rate of complications, and overall enhancement of hip function. In addition, the CCSBGS group performed better in respect of activities of daily living than the CCS, which more intuitively met patients' expectations of postoperative effects. Although no significant differences were found between the three groups in the number of people walking with or without crutches, the present study is preliminary in nature. Additional follow‐up studies with larger sample sizes are needed. At the same time, in future research, finite element analysis will be used to explore the biomechanical differences between CCS and CCSBGS.

## Author Contributions

**Peiyuan Wang:** conceptualization, methodology, software, data curation, investigation, validation, formal analysis, supervision, funding acquisition, visualization, project administration, resources, writing – review and editing, writing – original draft. **Zhiang Zhang:** conceptualization, methodology, software, data curation, supervision, formal analysis, validation, investigation, funding acquisition, visualization, project administration, resources, writing – review and editing, writing – original draft. **Zihang Zhao:** writing – review and editing. **Ziping Li:** writing – review and editing. **Lin Liu:** conceptualization. **Kuo Zhao:** writing – review and editing. **Lin Jin:** writing – review and editing. **Wei Chen:** writing – review and editing. **Shiqiang Zhang:** writing – review and editing. **Zhiyong Hou:** writing – review and editing.

## Ethics Statement

This retrospective study was approved by the Institutional Review Board of the 3rd Hospital of Hebei Medical University (G2020‐002‐1) before collecting data.

## Consent

The authors have nothing to report.

## Conflicts of Interest

The authors declare no conflicts of interest.

## Data Availability

The data that support the findings of this study are available from the corresponding author upon reasonable request.
